# Effects of an amino acid buffered hypochlorite solution as an adjunctive to air-powder abrasion in open-flap surface decontamination of implants failed for peri-implantitis: an ex vivo randomized clinical trial

**DOI:** 10.1007/s00784-022-04608-5

**Published:** 2022-07-08

**Authors:** Gerardo La Monaca, Nicola Pranno, Fabio Mengoni, Gianluca Puggioni, Antonella Polimeni, Susanna Annibali, Maria Paola Cristalli

**Affiliations:** 1 Department of Oral and Maxillofacial Sciences, University of Rome, 6. Caserta St, 00161 SapienzaRome, Italy; 2 Department of Public Health and Infectious Diseases, University of Rome, SapienzaRome, Italy; 3 Department of Molecular Medicine, “Sapienza” University Rome, Rome, Italy; 4 Department of Biotechnologies and Medical Surgical Sciences, University of Rome, SapienzaRome, Italy

**Keywords:** Biofilms, Dental implant, Air-powder abrasion, Chemical decontamination, Peri-implantitis, Microbiota

## Abstract

**Objectives:**

To evaluate ex vivo the efficacy of an amino acid buffered hypochlorite solution supplemented to surface debridement with air-powder abrasion in removing bacterial biofilm following open-flap decontamination of implants failed due to peri-implantitis.

**Materials and methods:**

This study was an ex vivo, single-blind, randomized, intra-subject investigation. Study population consisted of 20 subjects with at least three implants failed for peri-implantitis (in function for > 12 months and progressive bone loss exceeding 50%) to be explanted. For each patient, implants were randomly assigned to surface decontamination with sodium bicarbonate air-powder abrasion (test-group 1) or sodium bicarbonate air-powder abrasion supplemented by amino acid buffered hypochlorite solution (test-group 2) or untreated control group. Following open-flap surgery, untreated implants (control group) were explanted. Afterwards, test implants were decontaminated according to allocation and explanted. Microbiological analysis was expressed in colony-forming units (CFU/ml).

**Results:**

A statistically significant difference in the concentrations of CFU/ml was found between implants of test-group 1 (63,018.18 ± 228,599.36) (*p* = 0.007) and implants of test-group 2 (260.00 ± 375.80) (*p* < 0.001) compared to untreated implants (control group) (86,846.15 ± 266,689.44). The concentration of CFU/ml on implant surfaces was lower in test-group 2 than in test-group 1, with a statistically significant difference (*p* < 0.001).

**Conclusion:**

The additional application of amino acid buffered hypochlorite solution seemed to improve the effectiveness of implant surface decontamination with air-powder abrasion following open-flap surgery.

Clinical relevance.

Lacking evidence on the most effective method for biofilm removal from contaminated implant surfaces, the present experimental study provides further information for clinicians and researchers.

## Introduction

At the World Workshop on the Classification of Periodontal and Peri-Implant Diseases and Conditions, peri-implantitis has been defined as a pathological condition associated with plaque and characterized by the peri-implant mucosa inflammation response to the bacterial biofilm on the implant surface and the subsequent progressive loss of supporting bone [1]. Therefore, in peri-implantitis, implant surface decontamination removing plaque biofilm and calcified deposits is considered pivotal to either a reconstructive or resective surgical [2].

Various treatments have been proposed in the literature for implant surface decontamination during surgical procedures, including mechanical, chemical, photodynamic, and laser methods. However, no method has been shown to thoroughly remove peri-implant biofilm from contaminated implant surfaces [3–7]. The most common intraoperative surface treatment is mechanical debridement (manual, ultrasonic, sonic, or air-powder abrasive). When used alone, it failed to eliminate bacterial biofilm from infected implant surfaces even if showing a statistically significant difference than chemical decontamination [8–10].

Therefore, it has been proposed the additional use of antimicrobial treatments with local disinfectants or antibiotics such as delmopinol, chlorhexidine, cetylpyridinium chloride (CPC), tetracycline, minocycline, doxycycline, citric acid at pH 1, 3%, hydrogen peroxide, ethylenediamine tetra-acetate (EDTA), and 35% phosphoric acid gel [4, 6, 7]. As none of these substances has shown superior results, several adjunctive agents have been tested.

An amino acid buffered hypochlorite solution (Perisolv® De Ore biomaterials Negrar — Verona Italy) has been previously used for non-surgical periodontal and peri-implant therapy for the surface treatment of involved teeth and implants [11–14]. The product is a system with two components: sodium hypochlorite solution (NaOCl) at 0.95% and gel containing amino acids (glutamic acid, leucine, lysine), carboxymethylcellulose, and ultrapure water (pH > 10). The mixing of components contained in two syringes leads to the synthesis of the stable monochlorinated forms of the amino acids (named chloramines) able to alter the biofilm matrix [15, 16]. Furthermore, the different side-chain properties (positively charged, negatively charged, and hydrophobic) of the three chloroaminoacids enhance the antimicrobial activity, electrostatically attracting all proteins and large organic molecules. The specificity toward proteins introduced by amino acid chlorination gives potential protection for the mineralized tissue, and the high pH decreases the mineral structure solubility. At last, transferring at alkaline pH chlorine atoms to the amino acids exerts some protective effect against protein modification and cytotoxicity induced by hypochlorite, reducing the oxidant reactivity and making it less aggressive to healthy tissue [15–17]. Currently, this novel gel formulation’s effects on clinical parameters (clinical plaque index, probing depth, clinical attachment level, bleeding on probing) in the treatment of biofilm-associated peri-implant infections have been limited to non-surgical interventions. Therefore, the present ex vivo randomized clinical trial aimed to evaluate the efficacy of an amino acid buffered hypochlorite solution supplemented to surface debridement with air-powder abrasion in removing bacterial biofilm following open-flap decontamination of failed implants due to peri-implantitis. The null hypothesis was no statistically significant differences in the concentrations of colony-forming units (CFU/ml) between implants treated with air-powder abrasion vs adjunctive sodium hypochlorite gel buffered with amino acids.

## Materials and methods

### Study design

The trial was an ex vivo, single-blind, randomized, within-subject investigation comparing concentrations of colony-forming units (CFU/ml) on surfaces of contaminated implants treated with or without adjunctive amino acid buffered hypochlorite solution (chloramines gel) and explanted due to severe peri-implantitis. In the within-subject experimental design of the present study, each subject experienced the single implant treatments, including the control (untreated implants), removing subject-to-subject variation of different treatments. The study protocol was approved by the Department of Oral and Maxillofacial Sciences-Sapienza, University of Rome, Italy (Protocol identifying number: 0001558) and performed in accordance with the 1975 Declaration of Helsinki on medical protocols and ethics and its later amendments.

### Study population

Patients involved in the study were recruited between November 2019 and December 2020 from subjects referred to the Oral Surgery Unit, Policlinico Umberto I, “Sapienza” University of Rome, Italy, for treating peri-implantitis. Inclusion criteria were as follows: (1) at least three hopeless osseointegrated implants without no restriction regarding brands, types, and surfaces, functioning for > 12 months; (2) progressive bone loss exceeding 50% of the implant length detected on periapical radiographs; (3) presence of bleeding on gentle probing and/or suppuration. Exclusion criteria were as follows: (1) implant mobility; surgical or non-surgical peri-implant treatment carried out in the previous 6 months; antibiotic therapy assuming during the past 15 days.

All patients signed written informed consent after receiving detailed descriptions of the procedure. For each patient, implants were randomly assigned to surface decontamination with sodium bicarbonate air-powder abrasion (test-group 1) or sodium bicarbonate air-powder abrasion supplemented by amino acid buffered hypochlorite solution (test-group 2) or untreated control group. The randomization was performed with a list of random numbers generated using CLINSTAT software (Martin Bland, York, UK) and sequentially numbered opaque sealed envelopes. The surgeon carried out the decontamination method indicated in the envelope. The microbiologist assessor was unaware of the delivered treatment. The treatment code was not revealed until all microbiological tests had been completed and the data file had been established.

### Decontamination procedures

All procedures were performed by the same surgeon (G.L.M), experienced in treating implants with the diagnosis of peri-implantitis.

The protocol involved mouth wash rinses with 0.2% chlorhexidine digluconate solution (Corsodyl, GlaxoSmithKline Consumer Healthcare S.p.A. Baranzate, Milan, Italy) for 2 min, immediately before the intervention. After performing local anesthesia with 2% mepivacaine and 1:100,000 adrenalin (Carbocaine, AstraZeneca, Milan, Italy), the prosthetic supra-structure was removed, and a linear incision was made. Buccal and oral mucoperiosteal flaps were elevated, and granulation tissue was removed with titanium curettes (Hufriedy, Chicago, IL, USA) to expose implant surfaces. The operative field was irrigated with sterile saline solution for 1 min.

**Fig. 1:**
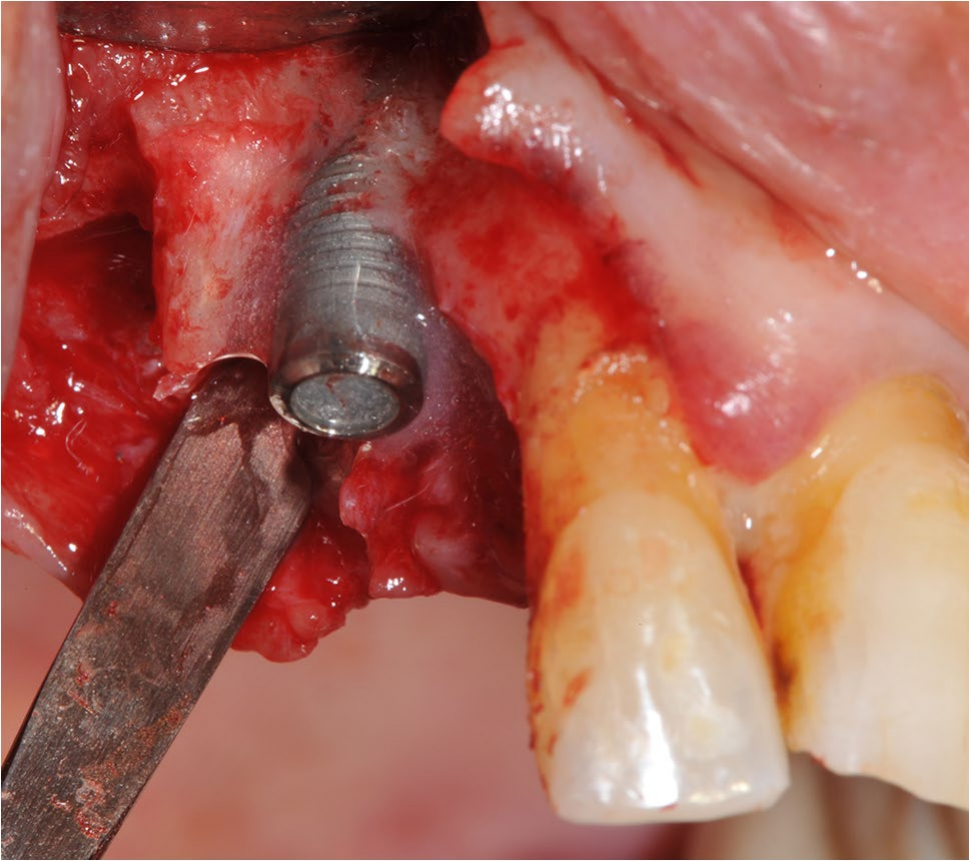
Amino acid buffered hypochlorite solution applied on implant surface after air-powder debriding with sodium bicarbonate

According to allocation, the control group implants were explanted untreated before performing the decontamination procedures for being used as positive control tests. Implants selected for test-group 1 were treated with sodium bicarbonate using a powered air-abrasion device (PROPHYflex™ 3 with periotip, KaVo, Biberach, Germany), rinsed with sterile saline solution, and then explanted. Implants of test-group 2 were first treated, applying the mixed gel on contaminated surfaces with a blunt cannula for 30 s and rinsing with sterile saline solution for 1 min. Subsequently, air-powder abrasion with sodium bicarbonate was performed. After rinsing with sterile saline solution for 1 min, decontamination with the chloramines gel was again carried out for 30 s (Fig. 1). At the end of treatments, implants of test-group 2 were rinsed for 1 min with sterile saline solution and explanted.

Explantation procedures in test and control groups were performed employing an implant retrieval kit (Implant Retrieval Kit–Nobel Biocare). Implants engaged with a specific tool adapter to the manual torque wrench were pulled out with anti-rotational movements and, to avoid contamination by oral cavity bacteria, directly transferred into one tube containing 1.5 ml of thioglycollate medium [14]. At the end of interventions, mucoperiosteal flaps were sutured with resorbable interrupted stitches (5–0 Vicryl, Ethicon S. p. A. Johnson & Johnson, Pratica di Mare, Rome, Italy), which were removed after 2 weeks. All patients received amoxicillin (875 mg) plus clavulanic acid (125 mg) (Augmentin, GlaxoSmithKline S.p.A., Verona, Italy) twice and metronidazole (250 mg) (Flagyl, Zambon, Milan, Italy) three times daily for 1 week and ketoprofen (Ibifen, Istituto Biochimico Italiano G. Lorenzini S. p. A., Aprilia, Latina, Italy) 200 mg for a maximum of three times daily according to individual post-operative pain.

### Microbiological sampling and analysis

Immediately after explantation, tubes containing the implants in 1.5 ml of thioglycollate medium were labelled with a code number and transferred to the microbiology laboratory. Within 4 h, tubes were vortexed for 90 s with a vortex mixer (VELP Scientifica) and then sonicated at a frequency of 40 kHz at 22 °C for 5–7 min (BANDELIN Electronic GmbH & Co. KG, Berlin, Germany) and finally vortexed again for 90 s to obtain a biofilm disintegration. The sonication fluid was centrifuged at 3200 rpm for 15 min, the supernatant was carefully removed, and the sediment was resuspended in 100 μl of medium. A 10-μl volume of medium was added onto aerobic Columbia sheep blood agar plates and incubated at 37 °C for 5 days. The same volume was placed onto anaerobic Schaedler sheep blood agar and incubated for 10 days in an anaerobic jar with CO_2_ generating system GasPak™ (Becton, Dickinson and Company). The semiquantitative estimate was made by counting on plates and expressed in colony-forming units (CFU/ml). The minimum detection level was 5 CFU/ml.

Microbial identification was performed by Bruker MALDI-TOF MS (Bruker Daltonics, Billerica, MA, USA), and for each implant, species found on plates were identified for multiple comparisons.

### Sample size calculation

The effect size value was calculated based on the mean concentrations of CFU/ml for each group evaluated in the first five patients (15 implants), using statistics software (GPower 3.1.9.2, Heinrich-Heine-Universität, Düsseldorf, Germany). A power analysis using the ANOVA test with three measurements, an alpha level of 0.05, and a medium effect size (*f* = 0.90) showed that 60 implants would be adequate to obtain 95% power in detecting a statistical difference in the CFU/ml between control and treatment groups assuming a loss of 20% of the sample during all procedures [18].

### Statistical analysis

The implant was chosen as the unit for the statistical analysis. Data were evaluated using standard statistical analysis software (version 20.0, Statistical Package for the Social Sciences, IBM Corporation, Armonk, NY, USA). A database was created using Excel (Microsoft, Redmond, WA, USA). Descriptive statistics including mean ± SD values and percentage were calculated for each variable: concentrations of CFU/ml and microbiological differences. The Shapiro–Wilk test was used to determine whether the data conformed to a normal distribution. As a nonparametric distribution of the concentrations of CFU/ml between three groups was found, a Kruskal–Wallis test was conducted to determine differences in the implant surface detoxification treatments. Pairwise comparisons were performed using Dunn’s procedure [19] with a Bonferroni correction. Adjusted *p* values were presented. Qualitative microbiological differences were evaluated with Fisher’s exact test, and the cut-off for statistical significance was *p* ≤ 0.05.

## Results

Twenty consecutive patients with at least three hopeless osseointegrated implants (10 males and 10 females; age 62 ± 4.413 years) were selected for the study. A total of 60 implants were randomized, 20 for each of the three groups. None of the implants was lost during decontamination procedures or the incubation period. No adverse events were reported among the study subjects during surgical procedures and post-operative.

### Microbiological analysis

**Fig. 2:**
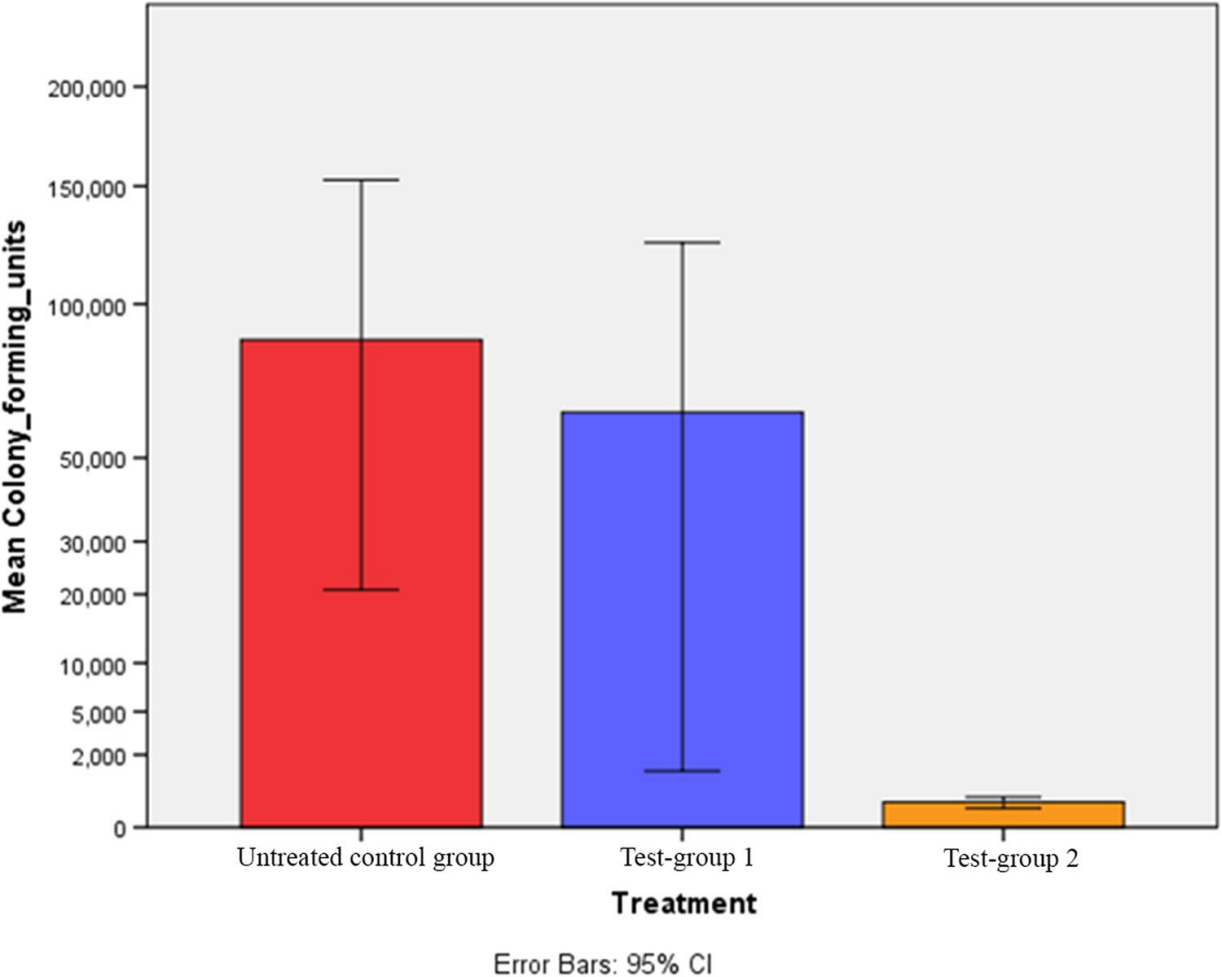
Simple bar chart based on the exponent (0.5) of mean CFU. The graph shows fewer CFU on implant surfaces of test-group 2 compared to those of test-group 1 or control group. The error bar represents the standard deviation of mean CFU/ml expressed using logarithmic notation

The mean value of concentrations of CFU/ml was 86,846.15 ± 266,689.44 for untreated implants (control group), 63,018.18 ± 228,599.36 for implants of test-group 1, decontaminated with air-powder abrasion alone, and 260.00 ± 375.80 for implants of test-group 2, decontaminated with air-powder abrasion supplemented by an amino acid buffered hypochlorite solution. The concentrations of CFU/ml for each of the three groups are illustrated in a bar chart showing the lowest value on implant surfaces of test-group 2 compared to those of test-group 1 and the control group (Fig. 2).

**Fig. 3:**
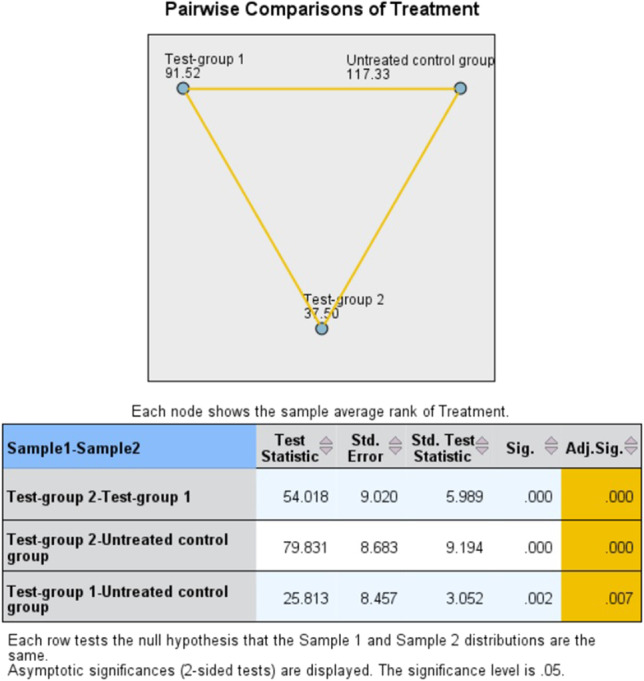
Diagram of pairwise comparison of decontamination methods: the numbers reflect the average rank for each group, and orange lines reflect a statistically significant pairwise comparison. Pairwise comparisons are detailed in the table below the diagram: the first column indicates which comparison is made and in what direction; the test statistic column reports the difference between mean ranks of the two groups; the Std. error and Std. test statistic columns present the standard error and the standardized statistic test, respectively; the Sig. and the Adj. columns show the unadjusted and adjusted *p* value, respectively

Pairwise comparisons with a Bonferroni correction for multiple comparisons revealed a statistically significant difference in the concentrations of CFU/ml between implants of the control group respect to implants of test-group 1 (*p* = 0.007) and implants of test-group 2 (*p* < 0.001) (Fig. 3). Furthermore, the statistically significant difference in the concentrations of CFU/ml between implants of test-group 1 and implants of test-group 2 (*p* < 0.001) proved that air-powder abrasion supplemented by an amino acid buffered hypochlorite solution was the most effective approach in implant surfaces decontamination (Fig. 3).

### Baseline microbiological findings

The microbiological analysis of all infected implants identified 14 microbial species without no statistically significant difference assessed by Fisher’s exact test (*p* = 0.824) in their distribution between test and control groups (Table 1). The most prevalent bacteria were *Neisseria subflavea* (20.6%), *Streptococcus parasanguinis* (11.8%), and *Streptococcus salivarius* (11.8%). Other species were present in less than 9% of the implants.

**Table 1:** Distribution of microorganism detected in control and test implants

Type of bacteria	Frequency (implants)	Percent	Valid percent	Cumulative percent
Strep. mitis/oralis	15	8.8	8.8	8.8
Strep. salivarius	20	11.8	11.8	20.6
Neisseria subflava	35	20.6	20.6	41.2
Staf. epidermidis	10	5.9	5.9	47.1
Enterococco faecalis	10	5.9	5.9	52.9
Staf. aureus	5	2.9	2.9	55.9
Neisseria flavescens	5	2.9	2.9	58.8
Strep. parasanguinis	20	11.8	11.8	70.6
Klebsiella oxytoca	5	2.9	2.9	73.5
Lactobacillus paracasei	15	8.8	8.8	82.4
Lactobacillus rhamnosus	10	5.9	5.9	88.2
Neisseria oralis	5	2.9	2.9	91.2
Lactobacillus spp.	5	2.9	2.9	94.1
Enterobacter aerogenes	10	5.9	5.9	100.0
Total	170	100.0	100.0	

## Discussion

In this ex vivo randomized clinical trial, the additional application of amino acid buffered hypochlorite solution seemed to improve the effectiveness of implant surface decontamination with air-powder abrasion in open-flap surgery. Therefore, the null hypothesis was rejected.

To the best of the authors’ knowledge, this is the first study to assess the adjunctive effect of amino acid buffered hypochlorite solution in bacterial biofilm removal from infected implant surfaces in surgical peri-implantitis treatment. Indeed, studies present in the literature about using this antiseptic solution are few and limited to evaluating the in vitro activity or clinical outcomes in non-surgical therapy of periodontitis, mucositis, and peri-implantitis.

Jurczyk et coll. [20] assessed the in vitro activity on bacteria associated with periodontitis of a sodium-hypochlorite formulation (NaOCl gel). NaOCl gel and its components sodium hypochlorite (NaOCl) and the activating vehicle (glutamic acid, leucine, lysine, carboxymethyl cellulose, and ultrapure water) were compared to 0.1% Chlorhexidine digluconate (CHX) solution and 0.9% sodium chloride (NaCl) as a positive and negative control, respectively. The antimicrobial activity was tested by determining minimal inhibitory concentrations (MICs), minimal bactericidal concentrations (MBCs), killing assays, and the influence on formation as well as on a 4-day-old 6-species biofilm. Generally, NaOCl gel had values of MICs higher than those of CHX solution but exerted a more growth inhibitory on Gram-negative than on Gram-positive bacteria. Although the active compound of NaOCl gel was mainly NaOCl, the activating vehicle itself had some activities on Gram-negative species, with a synergistic effect when NaOCl gel was compared with its compounds. Furthermore, biofilm formation was inhibited by CHX solution but not NaOCl gel. However, NaOCl gel, particularly its component NaOCl, performed a more remarkable activity in reducing the vitality of 4-day-old biofilm. This activity on the 4-day-old biofilm with the action against planktonic bacteria seemed to interfere with the biofilm matrix. The authors’ conclusions pointed to the potential of sodium-hypochlorite gel as an adjunctive topical antimicrobial treatment in mechanical therapy of periodontal disease.

The effect of an amino acid buffered hypochlorite solution on implant surface decontamination was also evaluated in vitro by Kubasiewicz-Ross et coll. [21]. In this study, 30 implants with different surfaces (machined, sandblasted and acid-etched, hydroxyapatite-coated) were coated with *Escherichia coli* biofilm, cultivated, and transferred in peri-implantitis jaw models with a 6-mm artificial bone defect. Implants were divided into 3 equal groups and treated with mechanical debridement with a sonic scaler device (1st group), mechanical debridement with sonic scaler applied for 2 min preceded by acid buffered hypochlorite solution left in situ for 30 s (2nd group) and Er: YAG laser irradiation (3rd group). The highest level of decontamination was achieved for machined-surface implants, and the mechanical debridement with sonic scaler supplemented with amino acid buffered hypochlorite solution and Er: YAG laser treatment.

Schmidlin et coll. [22] investigated the effects on bovine dentin discs of air polishing or amino acid buffered hypochlorite solution application on periodontal ligament (PDL) cell survival, attachment, and spreading. Human PDL cells were seeded onto the respectively treated discs and samples were then investigated for PDL cell survival, attachment, and spreading using a live/dead assay, adhesion assay, and SEM imaging. Significantly higher PDL cell numbers were found on samples treated either with air-polishing or rinsed with amino acid buffered hypochlorite solution compared to control samples (approximately 40% more cells; *p* < 0.05). Furthermore, SEM imaging revealed the potential for PDL cells to attach and spread on all surfaces. In vitro results of this study showed that cell survival and spreading of PDL cells are possible following amino acid buffered hypochlorite solution application.

Unlike the in vitro studies reporting the antimicrobial potential of amino acid buffered hypochlorite gel, all clinical trials found no statistically significant differences in the clinical outcomes of non-surgical mechanical debridement with or without gel adjunctive delivery.

In a randomized clinical trial, Roos-Jansaker et coll. [11 evaluated the adjunctive clinical effects of chloramine to non-surgical treatment of peri-implantitis. Plaque accumulation (Pl), probing depth (PD), clinical attachment level (CAL), and bleeding on probing (BoP) of 16 subjects with at least two implants with peri-implantitis were recorded at baseline and 3-month follow-up. The implants were randomized in test and control groups. Both implants received supramucosal and submucosal debridement by ultrasonic instrumentation supplemented with hand instruments. The implants assigned to the test group, before mechanical instrumentation, received local applications of a chloramine gel. Although, after 3 months, in both implant groups, a significant reduction (*p* < 0.001) in the number of BoP-positive sites compared with baseline was shown, no statistically significant differences for BoP or any other variables were found between the test and control groups. In conclusion, non-surgical mechanical debridement with adjunctive use of a chloramine is equally effective in reducing mucosal inflammation as conventional non-surgical mechanical debridement up to 3 months.

The clinical effects of amino acid buffered hypochlorite solution were also investigated by Iorio-Siciliano et coll. [12] in treating peri-implant mucositis in a 6-month randomized triple-blind controlled trial. Sixty-eight implants with mucositis were randomly assigned to two treatment groups. Before mechanical debridement with an ultrasonic scaler with a plastic tip, a sodium hypochlorite gel was delivered to the implants of the test group, while implants of the control group received a placebo gel. Both test and placebo gels were applied in the peri-implant sulcus for 30 s 5 times. The outcome variables were the change in pocket probing depth (PPD) and the number of implants with bleeding on probing (BoP) between baseline and 6 months. The 6-month results of that study, showing no statistically significant differences between the two groups with respect to evaluated outcome variables, indicated that mechanical debridement with adjunctive delivery of sodium hypochlorite gel was equally effective as non-surgical mechanical debridement alone.

In a randomized clinical trial of 12 months, Megally et coll. [13] evaluated the benefits of repeated short subgingival ultrasonic instrumentation with or without adjunctive administration of low-concentrated hypochlorite/amino acid gel in periodontal maintenance of pockets ≥ 5 mm. Thirty-two adult periodontal patients in maintenance care at least 3 months after periodontal therapy, with at least one residual periodontal pocket ≥ 5 mm, were randomly assigned to treatment by subgingival ultrasonic debridement with the gel or ultrasonic debridement only. In the test group, periodontal pockets were overfilled with hypochlorite/amino acid gel for 30 s before and after the instrumentation with an ultrasonic scaler for 1 min. In the control group, the treatment was limited to subgingival ultrasonic debridement. During 1-year maintenance visits, short ultrasonic instrumentation of residual pockets with PD ≥ 5 mm resulted in a clinically relevant CAL gain and PD reduction without further recessions. However, similar outcomes were reached whether delivering gel into periodontal pockets before and after repeated short ultrasonic debridement. Nevertheless, results indicated that the adjunctive use of the gel might be beneficial in deep residual pockets and sites with elevated bacterial counts, at least for a short time.

Conversely, statistically significant clinical improvement in terms of reduction of pocket depth at 6 to 12 months was found by Mayer et coll. [14] after adjunctive local antiseptic and anti-inflammatory therapy during a non-surgical mechanical treatment of peri-implantitis compared to ultrasonic debridement and soft tissue curettage with Teflon-coated curettes. The retrospective study involved 69 subjects with at least one titanium implant with peri-implantitis for a total of 106 implants. The tested procedure involved pockets filling with amino acid buffered hypochlorite solution for 30 s, soft tissue curettage, and mechanical debridement of implant surfaces with chitosan brushes. The hypochlorite and the curettage were repeated three times in the session before injection into the sulcus of microspheres containing 1 mg minocycline hydrochloride incorporated into a bioresorbable polymer.

Outcomes reported in human clinical trials mentioned above, referring to non-surgical treatment and clinical parameters, did not allow a direct comparison to findings of the present ex vivo study. Still, they might justify the biological rationale of the present investigation. Indeed, the failure of adjunctive amino acid buffered hypochlorite solution in non-surgical approaches may be due to limited access to bacteria laid down on infected implant surfaces. Conversely, bringing the mixed gel to direct contact with implant surfaces, such as in open-flap decontamination, would enhance the biofilm disruption assisting debridement with air-powder abrasion. The increased efficacy in removing bacterial deposits of the topical administration of amino acid buffered hypochlorite solution combined with mechanical treatment has been attributable to reducing the viability of bacterial species and altering the biofilm matrix. This antimicrobial activity, demonstrated by Jurczyk et al. [20] on bacteria associated with periodontitis, was shown effective also to the microbiota detected in the present investigation.

Furthermore, microbial species present on tested implants, only partially coincident with those found in an ex vivo study with a similar design [10], confirmed the complex and heterogeneous nature of the peri-implant infection often due to opportunistic pathogens [23–25].

Our findings on the benefits of adjunctive use of amino acid buffered hypochlorite solution in open-flap surface decontamination of implants with peri-implantitis were encouraging. Still, they must be interpreted with caution, considering the limitations and strengths of the present ex vivo investigation.

The first strength point was the use of implants to be explanted as a statistical analysis unit, which allowed within-subject evaluation. This experimental design, in which every participant is subjected to every single treatment, including the control, presents some advantages, such as not requiring a large pool of participants and helping reduce errors associated with individual differences. Then, individual differences in implant design and surface, and patient-related factors, such as oral hygiene level, peri-implant microbiota, history of periodontitis, or cigarette smoking, did not bias the treatment effects. The second strength point was the application of decontamination methods in actual clinical conditions, as treatment outcomes might be influenced by anatomical limitations of the oral cavity (e.g., the tongue) or the accessibility to infected implant surfaces. The main limitation was the lacking evaluation and comparison of decontamination procedures’ effectiveness on implants with different surface topographies, which might influence biofilm formation and removal. Another limitation was the semiquantitative analysis of the peri-implantitis microbiota, which is less sensitive than culture-independent techniques.

## Conclusions

In the present ex vivo randomized clinical trial, the additional application of amino acid buffered hypochlorite solution seemed to improve the efficacy of debridement with air-powder abrasion in removing bacterial biofilm from contaminated surfaces following surgical decontamination of implants with peri-implantitis. However, the positive effect in bacterial biofilm removal of the adjunctive use of amino acid buffered hypochlorite solution needs to be confirmed by additional in vitro studies and human clinical trials.
